# Graphene bilayer structures with superfluid magnetoexcitons

**DOI:** 10.1186/1556-276X-7-145

**Published:** 2012-02-21

**Authors:** Alexandr A Pikalov, Dmitrii V Fil

**Affiliations:** 1 Institute for Single Crystals, National Academy of Sciences of Ukraine, Lenin ave. 60, Kharkov 61001, Ukraine

**Keywords:** graphene, exciton superfluidity, multilayer heterostructures

## Abstract

In this article, we study superfluid behavior of a gas of spatially indirect magnetoexcitons with reference to a system of two graphene layers embedded in a multilayer dielectric structure. The system is considered as an alternative of a double quantum well in a GaAs heterostructure. We determine a range of parameters (interlayer distance, dielectric constant, magnetic field, and gate voltage) where magnetoexciton superfluidity can be achieved. Temperature of superfluid transition is computed. A reduction of critical parameters caused by impurities is evaluated and critical impurity concentration is determined.

## 1 Introduction

Recent progress in creation of heterostructures with two graphene layers separated by a thin dielectrics [1] opens possibilities to use graphene for creation of multiple quantum well structures with separately accessed conducting layers. In [1], SiO_2 _substrate and Al_2_O_3 _internal dielectric layer were used. Another promising dielectric is hexagonal BN [2]. It has a number of advantages, such as an atomically smooth surface that is free of dangling bonds and charge traps, a lattice constant similar to that of graphite, and a large electronic bandgap.

The attention to graphene heterostructures is caused, in some part, by the idea to use them for a realization of superfluidity of spatially indirect excitons [3-9]. Bound electron-hole pairs cannot carry electrical charge, but in bilayers they can provide a flow of oppositely directed electrical currents. Therefore, exciton superfluidity in bilayers should manifest itself as a special kind of superconductivity--the counterflow one, that means infinite conductance under a flow of equal in modulus and oppositely directed currents in the layers.

The idea on counterflow superconductivity with reference to electron-hole bilayers was put forward in [10,11]. The attempts to observe counterflow conductivity directly were done [12-14] for bilayer quantum Hall systems realized in GaAs heterostructures. In the latter systems superconducting behavior might be accounted for magnetoexcitons [15,16]. The effect is expected for the filling factors of Landau levels $\nu_{i}=2\pi \ell^{2}n_{i}(\ell =\sqrt{\hbar c/eB}$ is magnetic length, *n*_*i *_is the electron density in the *i*th layer) satisfying the condition *ν*_1 _+ *ν*_2 _= 1. The role of holes is played by empty states in zero Landau level. In experiments [12-14], an exponential increase of the counterflow conductivity under lowering of temperature was observed, but zero-resistance state was not achieved. The latter can be explained by the presence of unbound vortices [17-19]. Such vortices may appear due to spatial variation of the electron density caused by disorder.

To demonstrate counterflow superconductivity quantum Hall bilayers should have the parameters that satisfy two additional conditions: d≲ℓ and $\ell ≲a_{B}^{*}$, where *d *is the interlayer distance, and $a_{B}^{*}=\varepsilon \hbar^{2}/e^{2}m^{*}$ is the effective Bohr radius (*ε *is the dielectric constant of the matrix, and m* is the effective electron mass). The first inequality comes from the dynamical stability condition. For balanced bilayers (*ν*_1 _= *ν*_2_) the mean-fields theory yields *d *< 1.175 ℓ. The second inequality is the condition for the Coulomb energy *e*^2^/*ε*ℓ be smaller than the energy distance between Landau levels. In GaAs $a_{B}^{*}\approx 10\;\text{nm}$ and the condition $\ell ≲a_{B}^{*}$ is fulfilled at rather strong magnetic fields B≳6T (actually, the experiments [12-14] were done at smaller fields). At d≲10nm the interlayer tunneling is not negligible small and may result in a locking of the bilayer for the counterflow transport at small input current [20,21]. At larger input current the system unlocks, but the state becomes nonstationary one [22-24] that is accompanied by a dissipation (the power of losses is proportional to the square of the amplitude of the interlayer tunneling [22,24]).

The idea to use graphene for the realization of electron-hole superfluidity in quantum Hall bilayers [6-9] looks very attractive. The distance between Landau Levels in monolayer graphene is proportional to the inverse magnetic length, magnetic field does not enter into the condition of smallness of the Coulomb energy, and small magnetic fields can be used. Smaller magnetic fields correspond to smaller critical temperature, but, at the same time, they correspond to larger critical *d*. Use of large *d *allows to suppress completely negative effects caused by interlayer tunneling.

In this article, we concentrated on three questions. First, we determine, in what range of internal parameters and external fields magnetoexciton superfluidity can be realized. Second, we evaluate critical temperature for pure system. Third, we consider its reduction caused by electron-impurity interaction. Our study extends the results of [8], where a system of two graphene layers embedded into a bulk dielectric matrix was considered. Here we investigate structures with one and two graphene layers situated at the surface.

## 2 Conditions for the electron-hole pairing in zero Landau level

Quantum Hall effect in graphene is characterized by unusual systematics of Landau levels and the additional four-fold degeneracy connected with two valleys and two spin projections [25]. The energies of Landau levels in graphene are $E_{\pm N}=\pm \frac{\hbar v_{F}}{\ell }\sqrt{2\left|N\right|}$, where *N *= 0, 1, 2, ..., and *v*_*F *_≈ 10^6 ^m/s is the Fermi velocity. In a free standing graphene, the *N *= 0 Landau level is half-filled. A state with only completely filled Landau levels corresponds to a plateau at the Hall conductivity plot (dependence of *σ*_*xy *_on electron density). A free standing graphene is just between two plateaus [26]. A given quantum states in zero Landau level is characterized by the guiding center index *X *and the combination of the spin and valley indexes. Below we call four possible combinations, the components, and numerate them by the index *β *= 1, 2, 3, 4.

We describe electron-hole pairing in zero Landau level in graphene by the wave function that is a generalization of the wave function [15] to the multicomponent case

(1) $$|\Psi \rangle =\underset{\beta }{\prod^{\text{​}}}\underset{X}{\prod^{\text{​}}}(u_{\beta }c_{1\beta X}^{+}+v_{\beta }c_{2\beta X}^{+})|0\rangle .$$

Here $c_{i\beta X}^{+}$ is the electron creation operator (the operator that fills a given state in *N *= 0 Landau Level), |0〉 is the state with empty zero level, *i *is the layer index. The *u *- *v *coefficients satisfy the condition |*u*_*β*_|^2 ^+ |*v*_*β*_|^2 ^= 0. The function (1) can be rewritten in the form

(2) $$\left|\left(\Psi \right\rangle \right)=\underset{\beta }{\prod }\underset{X}{\prod }\left(u_{\beta }+v_{\beta }c_{2\beta X}^{+}h_{1\beta X}^{+}\right)\left|\left(vac1\right\rangle \right),$$

where $h_{1\beta X}^{+}=c_{1\beta X}$ is the hole creation operator, and the vacuum state is defined as $|vac1\rangle =\prod_{\beta }\prod_{X}c_{1\beta X}^{+}|0\rangle$. One can see that the function (2) is an analog of the BCS function in the Bardin-Cooper-Schrieffer theory of superconductivity.

The quantity ${\tilde{\nu }}_{\beta }={\left|u_{\beta }\right|}^{2}-{\left|v_{\beta }\right|}^{2}$ gives the filling factor imbalance for the component *β*. The order parameter of the electron-hole pairing reads as $\Delta_{\beta }=u_{\beta }^{*}v_{\beta }=\sqrt{1-{\tilde{\nu }}_{\beta }^{2}}e^{i\varphi }/2$. If a given component is maximally imbalanced $\left({\tilde{\nu }}_{\beta }=\pm 1\right)$ the order parameter *Δ*_*β *_is equal to zero.

If a one component bilayer system is balanced, the order parameter for the electron-hole pairing is maximum. But if the number of components is even, the balance $\sum_{\beta }{\tilde{\nu }}_{\beta }=0$ can be reached at ${\tilde{\nu }}_{\beta }=1$ for half of the components and ${\tilde{\nu }}_{\beta }=-1$ for the other half. In the latter case all *Δ*_*β *_= 0. As is shown below, just such a state corresponds to the energy minimum. In other words, in balanced graphene bilayers electron-hole pairing does not occur.

**Figure 1:**
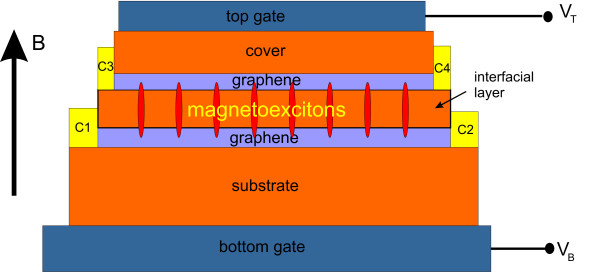
**Schematic view of the system under study. C1-C4 are the contacts**.

At nonzero imbalance $\sum_{\beta }{\tilde{\nu }}_{\beta }\neq 0,\pm 2,\pm 4$ at least for one component ${\tilde{\nu }}_{\beta }\neq \pm 1$, and electron-hole pairing may occur. Nonzero imbalance can be provided by electrical field directed perpendicular to the layers. Such a field can be created by a voltage difference applied between top and bottom gates (see, Figure 1).

We consider the general structure "dielectric 1-graphene 1-dielectric 2-graphene 2-dielectric 3" with three different dielectric constants *ε*_1_, *ε*_2_, and *ε*_3_. Dielectrics 1 and 3 are assumed to be thick (much thicker than the distance between graphene layers *d*). Solving the standard electrostatic problem we obtain the Fourier components of the Coulomb interaction *V*_*ii' *_for the electrons located in *i *and *i' *graphene layers

(3) $$V_{11}\left(q\right)=\frac{4\pi e^{2}}{q}\frac{\varepsilon_{2}+\varepsilon_{3}+\left(\varepsilon_{2}-\varepsilon_{3}\right)e^{-2qd}}{\left(\varepsilon_{2}+\varepsilon_{3}\right)\left(\varepsilon_{2}+\varepsilon_{1}\right)-\left(\varepsilon_{2}-\varepsilon_{3}\right)\left(\varepsilon_{2}-\varepsilon_{1}\right)e^{-2qd}},$$

(4) $$V_{22}\left(q\right)=\frac{4\pi e^{2}}{q}\frac{\varepsilon_{2}+\varepsilon_{1}+\left(\varepsilon_{2}-\varepsilon_{1}\right)e^{-2qd}}{\left(\varepsilon_{2}+\varepsilon_{3}\right)\left(\varepsilon_{2}+\varepsilon_{1}\right)-\left(\varepsilon_{2}-\varepsilon_{3}\right)\left(\varepsilon_{2}-\varepsilon_{1}\right)e^{-2qd}},$$

(5) $$V_{12}\left(q\right)=\frac{8\pi e^{2}}{q}\frac{\varepsilon_{2}e^{-qd}}{\left(\varepsilon_{2}+\varepsilon_{3}\right)\left(\varepsilon_{2}+\varepsilon_{1}\right)-\left(\varepsilon_{2}-\varepsilon_{3}\right)\left(\varepsilon_{2}-\varepsilon_{1}\right)e^{-2qd}}.$$

For electrons in *N *= 0 Landau level in graphene the Hamiltonian of Coulomb interaction has the form

(6) $$\begin{array}{l} H_{C}=\frac{1}{2S}\sum_{i,i^{\prime }}\sum_{X,X^{\prime }}\sum_{\beta ,\beta^{\prime }}\sum_{q}V_{ii^{\prime }}(q)e^{-\frac{q^{2}\ell^{2}}{2}+iq_{x}(X^{\prime }-X)}\\ c_{1\beta X+q_{y}\ell^{2}/2}^{+}c_{1^{\prime }\beta^{\prime }X^{\prime }-q_{y}\ell^{2}/2}^{+}c_{i^{\prime }\beta^{\prime }X^{\prime }+q_{y}\ell^{2}/2}c_{i\beta X-q_{y}\ell^{2}/2}, \end{array}$$

where *S *is the area of the system. The interaction with the gate field is described by the Hamiltonian

(7) $$H_{G}=-\frac{eV_{g}}{2}\underset{X\beta }{\sum }\left(c_{1\beta X}^{+}c_{1\beta X}-c_{2\beta X}^{+}c_{2\beta X}\right),$$

where *V*_*g *_is the interlayer voltage created by the external gate (bare voltage).

Rewriting the wave function (1) in the form

(8) $$|\Psi \rangle =\prod_{X}\prod_{\beta }\left(\cos\frac{\theta_{\beta }}{2}c_{1\beta X}^{+}+e^{i\varphi \beta }\sin\frac{\theta_{\beta }}{2}c_{2\beta X}^{+}\right)|0\rangle ,$$

and computing the energy in the state (8) we obtain

(9) $$E_{mf}=\frac{S}{8\pi \ell^{2}}\left(W\underset{\beta \beta^{\prime }}{\sum }\text{cos}\theta_{\beta }\text{cos}\theta_{\beta^{\prime }}-J_{0}\underset{\beta }{\sum }{\text{cos}}^{2}\theta_{\beta }-\left(2eV_{g}+J_{z}\right)\underset{\beta }{\sum }\text{cos}\theta_{\beta }\right),$$

where *W *= *e*^2^*d*/*ε*_2_ℓ^2 ^is the energy of direct Coulomb interaction. The exchange interaction energies

$$J_{ik}=\frac{1}{2\pi }\overset{\infty }{\underset{0}{\int }}qV_{ik}\left(q\right)e^{-\frac{q^{2}\ell^{2}}{2}}dq$$

determine the parameters *J*_0 _= (*J*_11 _+ *J*_22_)/2 - *J*_12 _and *J*_*z *_= *J*_11 _- *J*_22_. The relation between *θ*_*β *_and ${\tilde{\nu }}_{\beta }$ is given by equation ${\tilde{\nu }}_{\beta }=\text{cos}\theta_{\beta }$.

Taking into account the inequalities *W *>*J*_0_, and *J*_11_, *J*_22 _>*J*_12 _(that can be checked directly) we find that at *V*_*g *_= 0 the minimum of (9) is reached at ${\tilde{\nu }}_{1}={\tilde{\nu }}_{2}=1,{\tilde{\nu }}_{3}={\tilde{\nu }}_{4}=-1$. It indicates the absence of electron-hole pairing in balanced systems.

If *V*_*g *_≠ 0 and belongs to one of the intervals

(10) $$nW+J_{22}-J_{12}<eV_{g}<\left(n+2\right)W-J_{11}+J_{12},$$

where *n *= -4, -2, 0, 2, the energy minimum is reached at ${\tilde{\nu }}_{\beta_{a}}\neq \pm 1$ for one of the components. We will call such a component the active one.

Let us, for instance, consider the interval (10) with *n *= 0. Then the energy minimum is reached at

$${\tilde{\nu }}_{\beta_{a}}=\frac{eV_{g}+J_{z}/2-W}{W-J_{0}}.$$

The case ${\tilde{\nu }}_{\beta_{a}}=0$ (with maximum order parameter) corresponds to the voltage

(11) $$eV_{g}=-\frac{J_{z}}{2}+W.$$

Equation (11) determines the relation between magnetic field and the gate voltage *V*_*g*_. To keep ${\tilde{\nu }}_{\beta_{a}}=0$ the gate voltage should be varied synchronically with *B*. In particular, at *J*_*z *_= 0 (*ε*_1 _= *ε*_3_) the quantities *V*_*g *_and *B *are linearly related:

(12) $$V_{g}=\frac{\alpha dBc}{\varepsilon_{2}},$$

where *α *≈ 1/137 is the fine structure constant (the relation (12) is given in SI units).

If only the gate voltage or magnetic field is varied, the order parameter (and the critical temperature) changes nonmonotonically reaching the maximum at the point determined by (11).

## 3 Collective mode spectrum and phase diagram

The components that belong completely to one layer do not take part in the pairing. In what follows we consider the dynamics of only the active component.

We describe the active component by the wave function

(13) $$\left|\left(\Psi \right\rangle \right)=\underset{X}{\prod }\left(\text{cos}\frac{\theta_{X}}{2}c_{1,X+Q_{y}\ell^{2}/2}^{+}+e^{i\left(Q_{x}X+{\tilde{\varphi }}_{X}\right)}\text{sin}\frac{\theta_{X}}{2}c_{2,X-Q_{y}\ell^{2}/2}^{+}\right)\left|\left(0\right\rangle \right)$$

(here and below we omit the component index). Equation (13) describes the state with nonzero counterflow currents. To illustrate this statement we neglect for a moment the order parameter fluctuations $\left({\tilde{\varphi }}_{X}=0,\theta_{X}=\theta_{a}\right)$.

The order parameter is determined by the equation

(14) $$\Delta \left(r\right)=\underset{X,X^{\prime }}{\sum }\psi_{X}^{*}\left(r\right)\psi_{X^{\prime }}\left(r\right)\left\langle \Psi \left|c_{1,X}^{+}c_{2,X^{\prime }}\right|\Psi \right\rangle .$$

where

$$\psi_{X}\left(r\right)=\frac{1}{\pi^{1/4}\sqrt{\ell L_{y}}}e^{-i\frac{X_{y}}{\ell^{2}}}e^{-\frac{{\left(x-X\right)}^{2}}{2\ell^{2}}}$$

is the single-particle wave function in the coordinate representation, *L*_*y *_is the width of the system.

Substitution (13) into (14) yields

(15) $$\Delta \left(r\right)=\frac{\text{sin}\theta_{a}}{2}e^{-\frac{Q^{2}\ell^{2}}{2}}e^{iQ\cdot r}.$$

One can see from equation (15) that **Q **= (*Q*_*x*_, *Q*_*y*_) is the gradient of the phase of the order parameter.

Computing the energy in the state (13) and neglecting the fluctuations we obtain

(16) $$E_{0}=\frac{S}{8\pi \ell^{2}}\left(\left[W-F_{S}\left(0\right)\right]{\text{cos}}^{2}\theta_{a}-F_{D}\left(Q\right){\text{sin}}^{2}\theta_{a}\right),$$

where

(17) $$F_{S}\left(q\right)=\frac{1}{4\pi }\overset{\infty }{\underset{0}{\int }}pJ_{0}\left(pq\ell^{2}\right)\left[V_{11}\left(p\right)+V_{22}\left(p\right)\right]e^{-\frac{p^{2}\ell^{2}}{2}}dp,$$

and

(18) $$F_{D}\left(q\right)=\frac{1}{2\pi }\overset{\infty }{\underset{0}{\int }}pJ_{0}\left(pq\ell^{2}\right)V_{12}\left(p\right)e^{-\frac{p^{2}\ell^{2}}{2}}dp.$$

Electrical currents can be found from a variation of the energy caused by a variation of the vector-potential

(19) $$\delta E=-\frac{1}{c}\int d^{2}r\underset{i}{\sum }j_{i}\delta A_{i}.$$

Here **A**_*i *_is the in-plane component of the vector-potential in the layer *i*. To obtain the explicit expression for the variation (19) we replace the phase gradient in (16) with the gauge-invariant expression $Q-\frac{e}{\hbar c}\left(A_{pl,1}-A_{pl,2}\right)$, where **A**_*pl,i *_is the parallel to the graphene layers component of the vector potential in the layer *i*. Then, using (19) one finds the currents

(20) $$j_{1}=-j_{2}=-\frac{e}{\hbar }\frac{{\text{sin}}^{2}\theta_{a}}{8\pi \ell^{2}}\frac{dF_{D}\left(Q\right)}{dQ}.$$

At small gradients *Q*ℓ ≪ 1 equation (20) is reduced to

(21) $$j_{1}=\frac{e}{\hbar }\rho_{s0}Q,$$

where coefficient of proportionality between the current and the phase gradient

(22) $$\rho_{s0}=\frac{\ell^{2}}{32\pi^{2}}{\text{sin}}^{2}\theta_{a}\overset{\infty }{\underset{0}{\int }}p^{3}V_{12}\left(p\right)e^{-\frac{p^{2}\ell^{2}}{2}}dp$$

is called the zero temperature superfluid stiffness (the definition is given in the following section). Since we neglect fluctuations, the expression (20) yields the current at *T *= 0.

Implying the fluctuations of the amplitude and the phase of the order parameter are small one can present the energy as

(23) $$E=E_{0}+E_{2}+\ldots$$

The quadratic in fluctuations term can be diagonalized:

(24) $$E_{2}=\underset{q}{\sum }\left[m_{z}\left(-q\right)K_{zz}\left(q\right)m_{z}\left(q\right)+\frac{1}{4}\varphi \left(-q\right)K_{\varphi \varphi }\left(q\right)\varphi \left(q\right)-\frac{1}{2}\left(im_{z}\left(-q\right)K_{z\varphi }\left(q\right)\varphi \left(q\right)+c.c.\right)\right],$$

where

(25) $$\begin{array}{ll} m_{z}\left(q\right) & =\frac{1}{2}\sqrt{\frac{2\pi \ell^{2}}{S}}\underset{X}{\sum }\left(\text{cos}\theta_{X}-\text{cos}\theta_{a}\right)e^{-iqX},\;\\ \varphi \left(q\right) & =\sqrt{\frac{2\pi \ell^{2}}{S}}\underset{X}{\sum }\tilde{\varphi }\left(X\right)e^{-iqX}\;\\ & \; \end{array}$$

are the Fourier components of the fluctuations.

Equation (24) yields the energy of fluctuations with the wave vector directed along the *x *axis. The component of the matrix *K *can be presented in form independent of the choice of the direction of the coordinate axes

(26) $$K_{zz}\left(q,Q\right)=H\left(q,Q\right)-F_{S}\left(\left|q\right|\right)+F_{D}\left(\left|Q\right|\right)+\Xi \left(q,Q\right){\text{cot}}^{2}\theta_{a},$$

(27) $$K_{\varphi \varphi }\left(q,Q\right)={\text{sin}}^{2}\theta_{a}\Xi \left(q,Q\right),$$

(28) $$K_{z\varphi }\left(q,Q\right)=-\text{cos}\theta_{a}\left[F_{D}\left(\left|q+Q\right|\right)-F_{D}\left(\left|q-Q\right|\right)\right]/2,$$

where

(29) $$H\left(q,Q\right)=\frac{1}{2\pi \ell^{2}}\left[\frac{V_{11}\left(q\right)+V_{22}\left(q\right)}{2}-V_{12}\left(q\right)\text{cos}\left(\left|q\times Q\right|\ell^{2}\right)\right]e^{-\frac{q^{2}\ell^{2}}{2}}$$

(30) $$\Xi \left(q,Q\right)=\left[F_{D}\left(\left|Q\right|\right)-\frac{F_{D}\left(\left|q+Q\right|\right)+F_{D}\left(\left|q-Q\right|\right)}{2}\right].$$

The quantities *K*_*αβ*_(*q*) in (24) are expressed in terms of (26) as $K_{\alpha \beta }(q)=K_{\alpha \beta }(q,Q)|{}_{q=qi_{x}}.$.

The quantity *ħ *cos *θ *_*X*_/2 can be treated as a *z*-component of the pseudospin and it is canonically conjugated with the phase *φ*_*X*_. The Fourier transformed quantities (25) are defined as canonical variables as well. The equations of motion for the quantities *m*_*z*_(*q*) and *φ*(*q*) read as

(31) $$\hbar \frac{d\varphi \left(q\right)}{dt}=2K_{zz}\left(q\right)m_{z}\left(q\right)-iK_{z\varphi }\left(q\right)\varphi \left(q\right),$$

(32) $$\hbar \frac{dm_{z}\left(q\right)}{dt}=-\frac{1}{2}K_{\varphi \varphi }\left(q\right)\varphi \left(q\right)-iK_{z\varphi }\left(q\right)m_{z}\left(q\right).$$

Equation (31) yield the collective mode spectrum $\Omega \left(q,Q\right)=\sqrt{K_{\varphi \varphi }\left(q\right)K_{zz}\left(q\right)}+K_{z\varphi }\left(q\right)$. Rotating the axes one obtains the excitation spectrum at general **q**

(33) $$\Omega \left(q,Q\right)=\sqrt{K_{\varphi \varphi }\left(q,Q\right)K_{zz}\left(q,Q\right)+}K_{z\varphi }\left(q,Q\right).$$

At **Q **= 0 the spectrum (33) is isotropic. It can be presented in the Bogolyubov form

(34) $$\Omega_{0}\left(q\right)=\sqrt{\varepsilon_{q}\left(\varepsilon_{q}+\gamma_{q}\right).}$$

In equation (34)

(35) $$\varepsilon_{q}=F_{D}\left(0\right)-F_{D}\left(q\right)$$

is the kinetic energy (*ε*_*q *_≈ *ħ*^2^*q*^2^/2*M *at *q*ℓ ≪ 1, where *M *is the magnetoexciton mass, see, for instance [27]), and

(36) $$\gamma_{q}=\left[H\left(q,0\right)-F_{S}\left(q\right)+F_{D}\left(q\right)\right]{\text{sin}}^{2}\theta_{0}$$

has the sense of the exciton-exciton interaction energy (that includes the direct and exchange parts).

**Figure 2:**
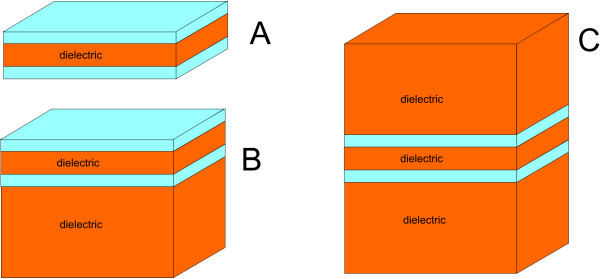
**Graphene heterostructures under study**.

The condition for the dynamical stability of the state (13) is the real valueness of the excitation spectrum (34). This condition determines the diapason of *d*/ℓ and *ε*_*i *_where superfluid magnetoexciton state can be realized. To be more concrete we consider three types of heterostructures. Type A is a graphene-dielectric-graphene sandwich with two graphene layers at the surface, Type B is a graphene-dielectric-graphene-dielectric structure with one such a layer, and Type C is a system of two graphene layers embedded in a dielectric matrix (Figure 2). For simplicity, we imply the same dielectric constants *ε *for the interfacial layer and the substrate.

**Figure 3:**
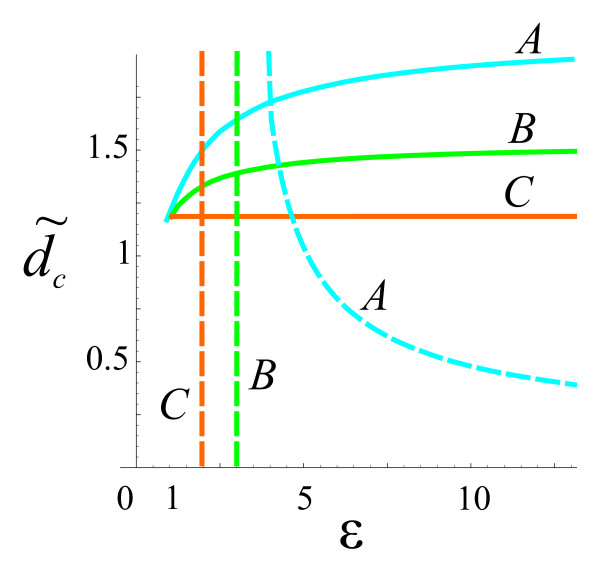
**Phase diagram at ${\tilde{\nu }}_{a}=0$ for the graphene bilayers of A, B, and C type. Solid curves, ${\tilde{d}}_{c}\left(\varepsilon \right)$; dashed curves, *ε***_***c***_**(*d*/ℓ)**.

The dynamical stability condition is fulfilled at $0<d/\ell <{\tilde{d}}_{c}\left(\varepsilon \right)$, where ${\tilde{d}}_{c}\left(\varepsilon \right)$ depends on the imbalance parameter ${\tilde{\nu }}_{\beta_{a}}\equiv {ṽ}_{a}$. The dependence ${\tilde{d}}_{c}\left(\varepsilon \right)$ at ${\tilde{\nu }}_{a}=0$ is shown in Figure 3.

The requirement for the Coulomb energy be smaller than the distance between Landau levels yields the restriction on *ε*. Since we study the pairing in *N *= 0 Landau level we compare the Coulomb energy with the energy distance between *N *= 0 and *N *= 1 levels $\omega_{c}=\sqrt{2}\hbar v_{F}/\ell$.

We have four parameters that characterize the Coulomb energy *W, J*_11_, *J*_22_, and *J*_12_. At $d/\ell <{\tilde{d}}_{c}$ the largest of them is *J*_11 _(the intralayer exchange interaction in the graphene layer at the surface). Therefore, it is natural to consider the condition

(37) $$J_{11}<\omega_{c}$$

as the additional restriction on the parameters. Equation (37) can be rewritten as *ε *>*ε*_*c*_(*d*/ℓ). The quantity *ε*_*c *_can be understood as a critical dielectric constant. The dependence *ε*_*c*_(*d*/ℓ) is also shown in Figure 3.

Two conditions $d/\ell <{\tilde{d}}_{c}\left(\varepsilon \right)$ and *ε *>*ε*_*c*_(*d*/ℓ) determine the range of parameters where one can expect a realization of electron-hole pairing and magnetoexciton superfluiduty in graphene bilayer systems.

## 4 Critical temperature

In a bilayer graphene heterostructure with a fixed *d *the magnetoexciton superfluidity can be realized in a wide range of magnetic field. Variation of *B *at fixed gate voltage results in a change of imbalance of the active component. Simultaneous tuning of *V*_*g *_allows to keep zero imbalance ${\tilde{\nu }}_{a}=0$ and maximum order parameter under variation of *B*. In this section, we study the dependence of critical temperature on magnetic field implying such a simultaneous tuning.

Superfluid transition temperature is given by the Berezinskii-Kostelitz-Thouless equation [15]

(38) $$T_{c}=\frac{\pi }{2}\rho_{s}\left(T_{c}\right),$$

where *ρ*_*s*_(*T*) is the superfluid stiffness at finite temperature. The superfluid stiffness is defined as the coefficient in the expansion of the free energy in the phase gradient $F=F_{0}+\int d^{2}r\rho_{s}{\left(\nabla \varphi \right)}^{2}/2$. In a weakly nonideal Bose gas it is equal to *ρ*_*s *_= *ħ*^2^*n*_*s*_/*m*, where *n*_*s *_is the superfluid density. As was shown in previous section, superfluid stiffness determines also the supercurrent.

Taking into account linear excitations we present the free energy *F *= *E*_0 _- *TS *in the following form

(39) $$F=E_{0}+T\underset{q}{\sum }\text{ln}\left(1-e^{\frac{-\Omega \left(q,Q\right)}{T}}\right).$$

Expansion of equation (39) yields the following expression for the superfluid stiffness

(40) $$\rho_{s}\left(T\right)=\rho_{s0}+\frac{1}{S}\underset{q}{\sum }\left[{\left(\left(\frac{d^{2}\Omega \left(q,Q\right)}{dQ^{2}}\right)\right|}_{Q=0}{\left(N_{q}-\frac{1}{T}N_{q}\left(1+N_{q}\right){\left(\frac{d\Omega \left(q,Q\right)}{dQ}\right)}^{2}\right|}_{Q=0}\right].$$

It follows from (40) and (33) that *ρ*_*s*_(*T*) <*ρ*_*s*0 _(thermal fluctuations reduce the superfluid stiffness).

For the spectrum *Ω*(**q**) = *E*(*q*) + *ħ***qv **(where **v **= *ħ*∇*φ*/*m *is the superfluid velocity) (40) yields the well-known answer for the superfluid density [28]. Equation (40) generalizes the results [28] for the general case.

**Figure 4:**
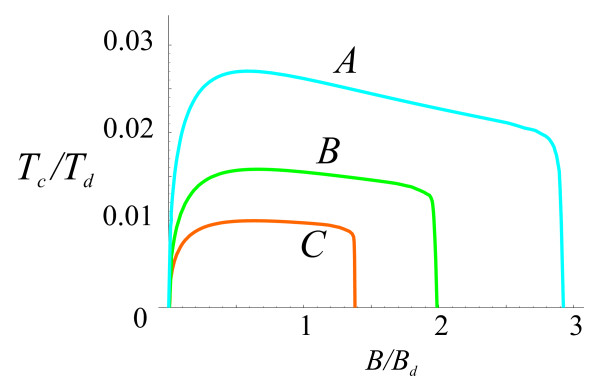
**Critical temperature vs magnetic field for A, B, and C structures. Temperature is given in units of *T***_***d ***_**= *e***^**2**^**/*εd*, magnetic field, in units of *B***_***d ***_**= *ϕ*/*πd***^**2**^.

The dependence of critical temperature on magnetic field at ${\tilde{\nu }}_{a}=0$ and *ε *= 4 is shown in Figure 4. One can see that the maximum critical temperature is reached approximately at *B *≈ 0.5*B*_*d*_, where *B*_*d *_= *ϕ*/*πd*^2 ^with *ϕ *= *hc*/2*e*, the magnetic flux quantum.

## 5 Influence of impurities on the critical parameters

In the previous section, we have determined the influence of thermal fluctuations on the superfluid stiffness. In this section, we consider the effect of reduction of the superfluid stiffness caused by the interaction of magnetoexcitons with impurities.

The Hamiltonian of the interaction of the active component with impurities can be presented in the form

(41) $$H_{\text{imp}}=\frac{1}{2S}\underset{q}{\sum }U_{z}\left(q\right)\left({\hat{\rho }}_{1}\left(q\right)-{\hat{\rho }}_{2}\left(q\right)\right),$$

where *U*_*z*_(**q**) = *U*_1_(**q**) - *U*_2_(**q**), *U*_*i*_(**q**) is the Fourier-component of the impurity potential in the layer *i*, and

(42) $${\hat{\rho }}_{i}\left(q\right)=\underset{X}{\sum }c_{i,X+\frac{q_{y}\ell^{2}}{2}}^{+}c_{i,X-\frac{q_{y}\ell^{2}}{2}}\text{exp}\left(-iq_{x}X-\frac{q^{2}\ell^{2}}{4}\right)$$

is the Fourier component of the electron density operator for the active component.

In the state (13), the energy of interaction with the impurities expressed in terms of *m*_*z*_(*q*) reads as

(43) $$E_{\text{imp}}=\text{const + }\underset{q}{\sum }{Ũ}_{z}\left(q\right)m_{z}\left(q\right),$$

where

$${Ũ}_{z}\left(q\right)=\frac{1}{\sqrt{2\pi \ell^{2}S}}U_{z}\left(qi_{x}\right)e^{-\frac{q^{2}\ell^{2}}{4}}.$$

The interaction (43) induces the fluctuations of the density and the phase of the order parameter.

Their values can be obtained from the Euler-Lagrange equations

(44) $$\begin{array}{c} \frac{\delta E}{\delta m_{z}\left(q\right)}=0,\\ \;\;\frac{\delta E}{\delta \varphi \left(q\right)}=0, \end{array}$$

where *E *is the energy of the system, described by the Hamiltonian *H *= *H*_*C *_+ *H*_*G *_+ *H*_imp _in the state (13).

Equations (44) solved in linear in impurity potential approximation yield

(45) $$m_{z}\left(q\right)=-\frac{1}{2}\frac{K_{\varphi \varphi }\left(q\right){Ũ}_{z}\left(q\hat{x}\right)}{K_{zz}\left(q\right)K_{\varphi \varphi }\left(q\right)-K_{z\varphi }^{2}\left(q\right)},$$

(46) $$\varphi (q)=\frac{iK_{z\varphi }(q){\tilde{U}}_{z}(q\hat{x})}{K_{zz}(q)K_{\varphi \varphi }(q)-K_{z}^{2}{}_{\varphi }(q)}.$$

Substituting (45), (46) into the expression for the energy one finds the correction to the energy caused by the electron-impurity interaction

(47) $$\Delta E=-\frac{1}{4}\underset{q}{\sum }{Ũ}_{z}\left(q\right){Ũ}_{z}\left(-q\right)\frac{K_{\varphi \varphi }\left(q\right)}{K_{zz}\left(q\right)K_{\varphi \varphi }\left(q\right)-K_{z\varphi }^{2}\left(q\right)}.$$

In equation (47), the contribution of fluctuations with the wave vectors directed along *x *is taken into account. Summing the contribution for all wave vectors one obtains

(48) $$\Delta E=-\frac{1}{8\pi \ell^{2}S}\underset{q}{\sum }U_{z}\left(q\right)U_{z}\left(-q\right)\frac{K_{\varphi \varphi }\left(q,Q\right)e^{-\frac{q^{2}\ell^{2}}{2}}}{K_{zz}\left(q,Q\right)K_{\varphi \varphi }\left(q,Q\right)-K_{z\varphi }^{2}\left(q,Q\right)}.$$

For simplicity, we specify the case where impurities are located in graphene layers. Then the Fourier-component of the impurity potential can be presented in the form

(49) $$U_{z}\left(q\right)=\underset{a}{\sum }e^{iqr_{{}_{a}}}u_{z,i}\left(q\right),$$

where **r**_*a *_are the impurity coordinates, and *u*_*z,i*_(**q**) = *u*_1,*i*_(**q**) - *u*_2,*i*_(**q**) with *u*_*k,i*_(**q**), the potential in the layer *k *of a single impurity centered at **r **= 0 in the layer *i*.

Averaging over impurities yields

(50) $$\Delta E=\frac{n_{\text{imp}}}{8\pi \ell^{2}}\underset{q}{\sum }\left({\left|u_{z,1}\left(q\right)\right|}^{2}+{\left|u_{z,2}\left(q\right)\right|}^{2}\right)\frac{K_{\varphi \varphi }\left(q,Q\right)e^{-\frac{q^{2}\ell^{2}}{2}}}{K_{zz}\left(q,Q\right)K_{\varphi \varphi }\left(q,Q\right)-K_{z\varphi }^{2}\left(q,Q\right)},$$

where *n*_imp _is the impurity concentration in a layer.

At *Q*ℓ ≪ 1 the energy (50) can be expanded in series as

(51) $$\Delta E=\Delta E_{0}+\frac{S}{2}\Delta \rho_{s}Q^{2},$$

where

(52) $$\Delta \rho_{s}=\frac{n_{\text{imp}}}{8\pi \ell^{2}S}\sum_{q}\frac{\left({\left|u_{z,1}(q)\right|}^{2}+{\left|u_{z,2}(q)\right|}^{2}\right)e^{-\frac{q^{2}\ell^{2}}{2}}}{K_{zz}^{2}(q,0)}\left({\frac{\partial^{2}K_{zz}(q,Q)}{\partial Q^{2}}|}_{Q=0}-\frac{2{\left(\frac{\partial K_{z\varphi }(q,Q)}{\partial Q}|{}_{Q=0}\right)}^{2}}{K_{\varphi \varphi }(q,0)}\right)$$

is the correction of the superfluid stiffness. One can check that the correction *Δρ*_*s *_is negative. Thus, the interaction with impurities results in decrease of critical parameters.

At ${\tilde{\nu }}_{a}=0$ equation (52) is reduced to

(53) $$\Delta \rho_{s}=-\frac{n_{\text{imp}}}{S}\underset{q}{\sum }\frac{\left({\left|u_{z,1}\left(q\right)\right|}^{2}+{\left|u_{z,2}\left(q\right)\right|}^{2}\right)e^{-\frac{q^{2}\ell^{2}}{2}}}{K_{zz}^{2}\left(q,0\right)}\left[\rho_{s0}-\frac{q^{2}V_{12}\left(q\right)e^{-\frac{q^{2}\ell^{2}}{2}}}{32\pi^{2}}\right].$$

where *ρ*_*s*0 _(equation (22)) is taken at *θ*_*a *_= *π*/2.

**Figure 5:**
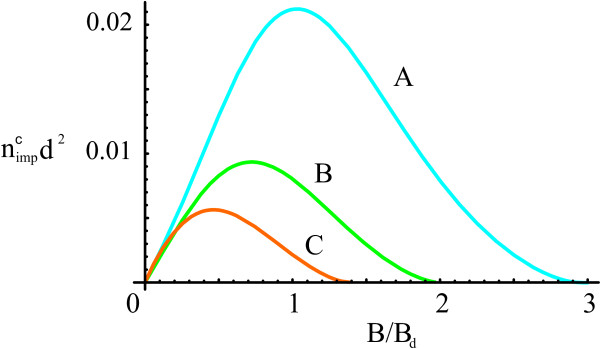
**Critical impurity concentration versus magnetic field for charged impurities located in graphene layers**.

The shift of critical temperature is evaluated as *ΔT*_*c*_/*T*_*c *_≈ *Δρ*_*s*_/*ρ*_*s*0_.^a ^We define the critical impurity concentration $n_{\text{imp}}^{c}$ as a concentration at which *Δρ*_*s*_/*ρ*_*s*0 _= 1. We consider charged impurities with the potential *u*_*z,i*_(**q**) = (-1)^*i*^(*V*_12_(**q**) - *V*_*ii*_(**q**)). The dependence of critical impurity concentration on magnetic field at *ε *= 4 and ${\tilde{\nu }}_{a}=0$ is shown in Figure 5. We also evaluated critical concentrations for neutral impurities. These concentrations are much larger, and the influence of neutral impurities can be neglected.

## 6 Conclusion

In conclusion, we present some estimates. Let us specify the type B structure (the one used in [1]) with *d *= 20 nm and *ε *= 4. For this structure the maximum critical temperature *T*_*c *_≈ 3 K (in pure case) is reached in magnetic field *B *≈ 0.8 T. At such *B *the critical impurity concentration is $n_{\text{imp}}^{c}\approx 2\cdot 10^{9}\text{c}{\text{m}}^{\text{ - 2}}$. The gate voltage determined by equation (11) is *V*_*g *_≈ 6 mV, that corresponds to electrostatic field *E *≈ 3 kVcm^-1^.

Basing on the results of our study we may state the following.

1. Graphene bilayer structures are perspective objects for the observation of magnetoexciton superfluidity. The advantages are smaller magnetic fields and no restriction from above on physical interlayer distance, that means the possibility to suppress completely interlayer tunneling.

2. Gate voltage should be created between graphene layers for a realization of magnetoexciton superfluidity.

3. Certain conditions on dielectric constant and on the ratio between interlayer distance and magnetic length should be satisfied.

4. Structures with graphene layers situated at the surface have larger critical parameters.

5. Neutral impurities are not dangerous for the magnetoexciton superfluidity, but the concentration of charged impurities should be controlled.

## Competing interests

The authors declare that they have no competing interests.

## Authors' contributions

AAP carried out the calculation and took part in the manuscript preparation. DVF designed and coordinated of the study and prepare the manuscript. All authors read and approved the final manuscript.

## Endnote

^a^Since in our approach we assume smallness of *Δp*_*s*_/*p*_*s*0 _it is just an estimate.
